# Immune Checkpoint Inhibitors: Think Beyond Cardiotoxicity! Is Physical Deconditioning a New Marker of Disease?

**DOI:** 10.1111/echo.70179

**Published:** 2025-05-30

**Authors:** Roberto de Castro Meirelles de Almeida, Aurora Felice Castro Issa, Alex dos Santos Felix

**Affiliations:** ^1^ Echocardiography Laboratory National Institute of Cardiology Rio de Janeiro Brazil; ^2^ National Institute of Cardiology Rio de Janeiro Brazil

1

Immune checkpoint inhibitors (ICI) are molecules that target and potentiate the immune response to cancer cells. In particular, they block programmed cell death protein 1 (PD‐1) and its ligand PD‐L1, as well as blocking cytotoxic T‐lymphocyte antigen (CTLA)‐4. They have brought notable advances in the treatment of advanced‐stage neoplasms, notably metastatic and inoperable melanomas [1].

The elegant and innovative article by Tsai, K. et al. [2] followed a cohort of patients with advanced, metastatic and/or inoperable melanoma, undergoing treatment with ICI and with a perception of fatigue, validated by established scores (Common Terminology Criteria for Adverse Events criteria and FACIT‐Fatigue subscale scores), comprehensively assessed by echocardiogram, various parameters of ventricular structure and function, including global and atrial longitudinal strain (speckle‐tracking), as well as physiological parameters of exercise such as peak physical exertion and maximum heart rate obtained at peak exertion, with baseline data recorded (prior to treatment initiation) and after 3 and 6 months.

Interestingly, at the end of the follow‐up period, there was no association between cardiotoxicity and the presence of fatigue documented by clinical questionnaires and the reduction in maximum exercise tolerance. No statistically significant changes were observed in cavity volumes and left and right ventricular function (with an isolated trend towards an increase in LV mass), nor in the results of global and atrial longitudinal strain or in diastole.

However, the perception of fatigue was indeed proven and there was a statistically significant reduction in stroke volume assessed by the velocity‐time integral of the LV outflow tract and the peak heart rate reached at maximum effort, leading us to hypothesize that, even in the absence of classic cardiotoxicity parameters already established in the literature [3, 4], the presence of fatigue correlated with reduced exercise tolerance and stroke volume.

We encourage and look forward to the follow‐up of this cohort, the inclusion of patients with other malignant neoplasms using ICI [5], more racial and gender diversity, and, if possible, the reproduction of this hypothesis in a multicenter setting. Recognizing, of course, the difficulty in selecting and monitoring patients with advanced cancer.

The use of serum biomarker dosage, especially natriuretic peptides and ultrasensitive troponin, and cardiopulmonary exercise testing, as well as correlation with different tissue assessment methods should bring even more information to this exciting field of discoveries in an area that is still little explored, that of clinical, laboratory and advanced imaging methods correlation in patients undergoing new molecular treatments, such as immunological and genetic targets. With an emphasis on measuring physiology and tolerance to physical exertion, which correlates with quality of life, independence, and even prognosis for this group of patients [6, 7]. Also, remembering the careful analysis of confounding factors for fatigue and those inherent in the progression of cancer itself, such as anemia, nutritional deficits, among others.

This could reinforce the association between clinically assessed fatigue and poor adaptation to exercise, as important factors impacting the use of ICI, constituting new therapeutic targets for intensive lifestyle modification, the development of new drugs, and directing the use of regimens already recommended for these patients.
